# Dietary source of polyunsaturated fatty acids influences cell cytotoxicity in broiler chickens

**DOI:** 10.1038/s41598-021-89381-3

**Published:** 2021-05-17

**Authors:** Hanan Al-Khalaifah, Afaf Al-Nasser

**Affiliations:** Environment and Life Sciences Research Center, Kuwait Institute for Scientific Research (KISR), Kuwait, Kuwait

**Keywords:** Biological techniques, Immunology, Zoology, Diseases, Health care

## Abstract

The current study aims to investigate the effects of dietary source of n-3 polyunsaturated fatty acids (PUFA) on immune response in broiler chickens, represented by cytotoxic cell activity. A total of 255 one-day-old male Cobb 500 broiler chickens were fed on fish oil (FO)-, flaxseed oil-enriched diets at 50 and 19 g/kg, respectively, in addition to the soybean-based control diet. At slaughter, samples of blood and spleen were harvested from 20 birds/treatment (n = 20). The immune tissues' fatty acid profile was analyzed by gas chromatography, and the cytotoxic cell activity was investigated. The results showed that supplementing broiler chickens with diets rich in n-3 PUFA had a substantial effect on the broiler immune tissues' fatty acid profile. Cytotoxic cell activity was significantly higher in splenocytes and peripheral blood mononuclear cells (PBMCs) from broilers fed flaxseed oil than those provided FO and the soybean control diet. These results suggest that flaxseed oil may be used to enrich chickens with n-3 PUFA and improve the immune status of chicken flocks to resist diseases.

## Introduction

N-3 polyunsaturated fatty acids (PUFA) are essential for general health in both humans and animals. The uptake of n-3 PUFA by human beings is low in modern societies. Therefore, researchers have enriched poultry meat with these beneficial fatty acids for healthier lifestyles^1–10^. These fatty acids have health importance because they influence living cells' vitality by incorporating them into the phospholipid layer in all biological cells. Two members of the n-3 PUFA family, eicosapentaenoic acid (EPA) and docosahexaenoic acid (DHA), compete with arachidonic acid (AA) for desaturase enzymes and eicosanoid production. Accordingly, this incorporation will lead to down-regulation of the inflammatory response of diseases related to cardiovascular, nervous, and digestive systems^2,11–19^. Recently, there has been some argument on the effect of PUFA on chicken's immune system. Some studies show no effect^20^, some offer a negative impact^21–23^, while some show an enhancement^20,24–27^.

The influence of dietary PUFAs on the immune system has been studied for decades. The coordination of the different immune cells and their activity is important for achieving a healthy immune system. This requires secretion of cytokines and chemokines. The cells from the innate immune stem include macrophages, neutrophils, eosinophils, basophils, mast cells, natural killer cells and dendritic cells whereas cells of the adaptive immune system are the B cells and T cells. Some of the immune cells are cytotoxic cells such as Natural Killer (NK) cells and T-cytotoxic cells, these are activated by the interaction of T cell receptor with antigen presenting cells like macrophages or dendritic cells. The effects of dietary omega-3 fatty acids on the abundance of T cells is not clear till date^28^. NK cells are lymphocytes involved in the innate and adaptive immunity. They kill unhealthy cells, secrete soluble factors and regulate response of antigen presenting cells and adaptive T cells. NK cells express certain receptors that activate their cytotoxic and secretory functions. NK cell cytotoxicity is strictly controlled by MHC-1-specific inhibitory receptors^29^. Till date there exists very limited information on the effects of PUFAs on cytotoxic cell activity. In mouse, it was reported that DHA activated splenic NK cells whereas in humans dietary supplementation with EPA had no impact on NK cells^28^.

Overall, some studies demonstrate that n-3 PUFA may suppress the NK cell activity in mice^30,31^, rats^32,33^, and humans^34,35^. These cells are responsible for killing tumor cells and cells infected with viruses^36^. However, there is limited research regarding the effect of n-3 PUFA on the activity of cytotoxic cells in chickens, including NK cells. Accordingly, this research aimed to explore the influence of feeding broiler chickens on diets rich in fish oil (FO) or flaxseed oil on cytotoxic cell activity in splenocytes and PBMCs of 5 wks old broiler chickens.

Traditionally, broiler diets lack n-3 PUFAs due to higher n-6:n-3 ratios, which could adversely affect the performance, immune system, and meat quality of birds. As a result, poultry researchers worldwide are making efforts to develop n-3 rich poultry products. The amount of n-3 PUFA in the animal tissue is mainly determined by the diet's fatty acid composition. Fish oil is a rich source of EPA and DHA, which are precursors of lipid mediators of inflammation. In contrast, plant-based oils are rich in ALA, which is the precursor for EPA and DHA, as was seen in the case of a study by Ibrahim et al.^37^, in their research using various sources of plant and animal oils such as FO and linseed oil. The diets were formulated with these dietary oil sources to study the effect of altering dietary n-6:n-3 PUFA and monitor its impact on performance, behavior, cytokine mRNA expression, anti-oxidative status, and meat fatty acid profile. Their results suggested that lowering very high n-6:n-3 PUFA ratios in broiler diets could improve their performance and immunity without compromising behavior. There were changes observed in meat fatty acid composition; the promising effects of reducing these PUFA ratios were more visible in broilers supplemented with FO than linseed oil. Sadeghi et al.^38^ reported in their study investigating the use of FO, soybean oil, and olive oil on performance and immune response in broiler chickens. The authors observed that soybean oil contributed to higher body weight gain and feed conversion ratio, whereas FO contributed to an overall improvement in broiler chickens' immune responses.

## Results

### Production performance parameters of broilers

The body weight, feed consumption, feed efficiency, and weekly body weight gain are shown in Tables 1, 2, 3, and 4, respectively. These tables show no significant differences in body weight, feed consumption, feed efficiency, and weekly body weight between broiler chickens fed different dietary treatments.

**Table 1:** The effect of different supplemental oils on body weight at different ages of broilers.

Age	Control	Body weight (g)		
		FLO	FO	*P *value
1 day	43.68 ± 2.01	44.4 ± 2.16	46.0 ± 1.03	0.117
1 week	130.72 ± 4.64	130.12 ± 7.40	128.98 ± 3.49	0.107
2 week	327.20 ± 15.7	325.96 ± 16.70	340.76 ± 11.04	0.504
3 week	595.0 ± 18.42	601.6 ± 26.36	603.92 ± 23.37	0.296
4 week	964.00 ± 41.2	943.20 ± 40.83	940.0 ± 30.65	0.454
5 week	1450.0 ± 54.2	1444.80 ± 39.3	1523.60 ± 132.4	0.245

Values are means ± SD.

*FLO* flaxseed oil, *F* fish oil.

**Table 2:** The effects of different supplemental oils on the feed consumption at different ages of broilers.

Age (weeks)	Feed consumption (g)			
	Control	FLO	FO	*P *value
1	114.36 ± 4.39	118.24 ± 2.27	116.16 ± 10.32	0.701
2	242.92 ± 4.59	241.46 ± 2.45	244.72 ± 3.24	0.195
3	482.48 ± 9.20	485.26 ± 8.25	482.34 ± 8.67	0.886
4	622.76 ± 23.01	642.72 ± 31.29	629.38 ± 29.31	0.973
5	892.93 ± 138.18	900.6 ± 174.58	901.0 ± 177.75	1.000
Total feed (g)	2354.12 ± 172.95	2399.68 ± 159.70	2386.66 ± 189.97	0.975

Values are means ± SD.

*FLO* flaxseed oil, *FO* fish oil.

**Table 3:** The effects of different supplemental oils on the feed efficiency at different ages of broilers.

Age (weeks)	Feed efficiency			
	Control	FLO	FO	*P *value
1	1.32 ± 0.08	1.38 ± 0.08	1.41 ± 0.21	0.080
2	1.24 ± 0.08	1.23 ± 0.06	1.16 ± 0.06	0.534
3	1.81 ± 0.17	1.77 ± 0.19	1.85 ± 0.15	0.581
4	1.71 ± 0.24	1.90 ± 0.21	1.90 ± 0.28	0.146
5	1.88 ± 0.47	1.82 ± 0.44	1.66 ± 0.61	0.920
Overall	1.62 ± 0.21	1.66 ± 0.11	1.58 ± 0.21	0.443

Values are means ± SD.

*FLO* flaxseed oil, *FO* fish oil.

**Table 4:** The effects of different supplemental oils on the weekly body weight gain at different ages of broilers.

Age (weeks)	Weekly body weight gain (g)			
	Control	FLO	FO	*P *value
1	87.04 ± 5.87	85.72 ± 6.43	82.96 ± 6.30	0.118
2	196.48 ± 11.69	195.84 ± 9.30	211.8 ± 12.03	0.287
3	264.8 ± 21.77	275.72 ± 25.68	263.16 ± 24.72	0.532
4	369.00 ± 49.80	341.52 ± 41.24	336.08 ± 56.11	0.378
5	488.00 ± 89.84	501.60 ± 37.16	583.60 ± 152.40	0.337
Total body weight gain	1408.32 ± 55.29	1400.40 ± 38.57	1477.60 ± 134.44	0.374

Values are means ± SD.

*FLO* flaxseed oil, *FO* fish oil.

### Fatty acid profile of immune cells

Table 5 shows the effect of feeding 5-wk-old broiler chickens n-3 PUFA sources on the fatty acid profile of splenocytes and blood leukocytes. Supplementing broilers with diets rich in FO resulted in significantly higher enrichment of EPA (C20:5n3) and DHA (C18:2n-6) in splenocytes than those fed flaxseed oils and the control group (Table 5). On the other hand, α-linolenic acid (C18:3n3) was higher in broilers fed flaxseed oil than those provided FO and the control group. There was no significant difference in SDA concentrations (C18:4n3) between broilers fed different experimental treatments. The total n-3 PUFA were significantly higher in broilers fed FO than those fed flaxseed oils and the control group (Table 5). AA (C20:4n6) in spleens from broilers fed flaxseed oil and the control group was significantly higher than that for broilers fed FO. Spleens of broilers fed the control diet and flaxseed oil showed similar total proportions of n-6 PUFA, which were more significant than those supplemented with FO. FO feeding resulted in a significantly lower ratio of n-6:n-3 than feeding flaxseed oil and the control diet. The same trend was observed for the fatty acid profile of the broilers' PMBCs fed the different diets (Table 5).

**Table 5:** Fatty acid composition of spleen and blood leukocytes of 5-wk-old broilers fed the experimental diet.

	% of the total weight				
	Control	FO	Flaxseed	SEM	*P *value
**Spleen**					
C18:2n6 (linoleic)	18.00^a^	11.76^b^	17.00^a^	0.699	< 0.001
C18:3n-6(γ-linolenic)	0.96	1.00	2.00	0.612	0.199
C18:3n3 (α-linolenic)	1.99^c^	0.87^ab^	1.00^bc^	0.090	< 0.001
C18:4n3(stearidonic acid)	0.23	1.01	0.38	0.199	0.515
C20:2n-6 (eicosadienoic)	3.00^a^	1.67^b^	2.80^a^	0.100	< 0.001
C20:3n-6	4.01^ab^	3.01^a^	5.57^a^	0.299	0.008
C20:4n6 (arachidonic)	16.00^a^	9.01^b^	14.33^a^	1.55	0.011
C20:5n3 (EPA)	0.87^a^	9.02^c^	5.87^b^	0.303	< 0.001
C22:5n3 (DPA)	4.00^c^	6.20^b^	5.50^ac^	0.400	< 0.001
C22:6n3 (DHA)	2.70^b^	7.11^c^	4.70^b^	0.311	< 0.001
∑n-6	41.97^a^	26.45^b^	41.70^a^	1.989	< 0.001
∑n-3	9.79^b^	24.21^c^	17.45^b^	0.621	< 0.001
∑n-6:∑n-3	4.29^b^	1.09^a^	2.38^b^	0.323	< 0.001
**PBMCs**					
C18:2n6	13.72^b^	5.89^c^	14.10^b^	0.800	< 0.001
C18:3n-6	0.78^b^	0.31^a^	0.74^b^	0.198	0.020
C18:3n3	2.27^c^	0.44^ab^	1.18^b^	0.201	0.041
C18:4n3	0.48	0.32	0.22	0.200	0.477
C20:2n-6	1.11^c^	0.39^b^	0.69^a^	0.033	< 0.001
C20:3n-6	3.47	2.65	3.21	0.601	0.722
C20:4n6	9.33^b^	3.86^c^	9.51^b^	0.199	< 0.001
C20:5n3	1.01^b^	7.11^c^	2.91^b^	0.399	< 0.001
C22:5n3	3.15^a^	5.18^b^	5.71^a^	0.400	< 0.001
C22:6n3	1.63^b^	6.94^c^	3.48^b^	0.301	< 0.001
∑n-6	28.41^b^	13.10^c^	28.25^b^	0.989	< 0.001
∑n-3	8.54^b^	19.99^a^	13.50^b^	0.820	< 0.001
∑n-6:∑n-3	3.33^b^	0.66^a^	2.09^b^	0.502	< 0.001

Means within rows with no common superscripts are significantly different (P ≤ 0.05), one-way analysis of variance (ANOVA) and the general linear model procedure of Minitab was applied. Treatment mean differences were identified by Bonferroni tests, values are expressed as means (n = 20, 20 birds per each dietary treatment), pooled standard error of means (SEM), *C18:2n6* linoleic, *C18:3n-6* γ-linolenic, *C18:3n3* α-Linolenic, *C18:4n3* stearidonic acid, *C20:2n-6* eicosadienoic, *C20:4n6* arachidonic, *C20:5n3* eicosapentaenoic acid (EPA), *C22:5n3* docosapentaenoic acid (DPA), *C22:6n3* docosahexaenoic acid(DHA), *∑n-6* Sum percentage of n-6 PUFA, *∑n-3* sum percentage of n-3 PUFA, *∑n-6:∑n-3* ratio of ∑n-6 to ∑n-3, *FO* fish oil, *PBMCs* peripheral blood mononuclear cells.

### Splenocyte and PBMCs cytotoxic cell activity

**Figure 1:**
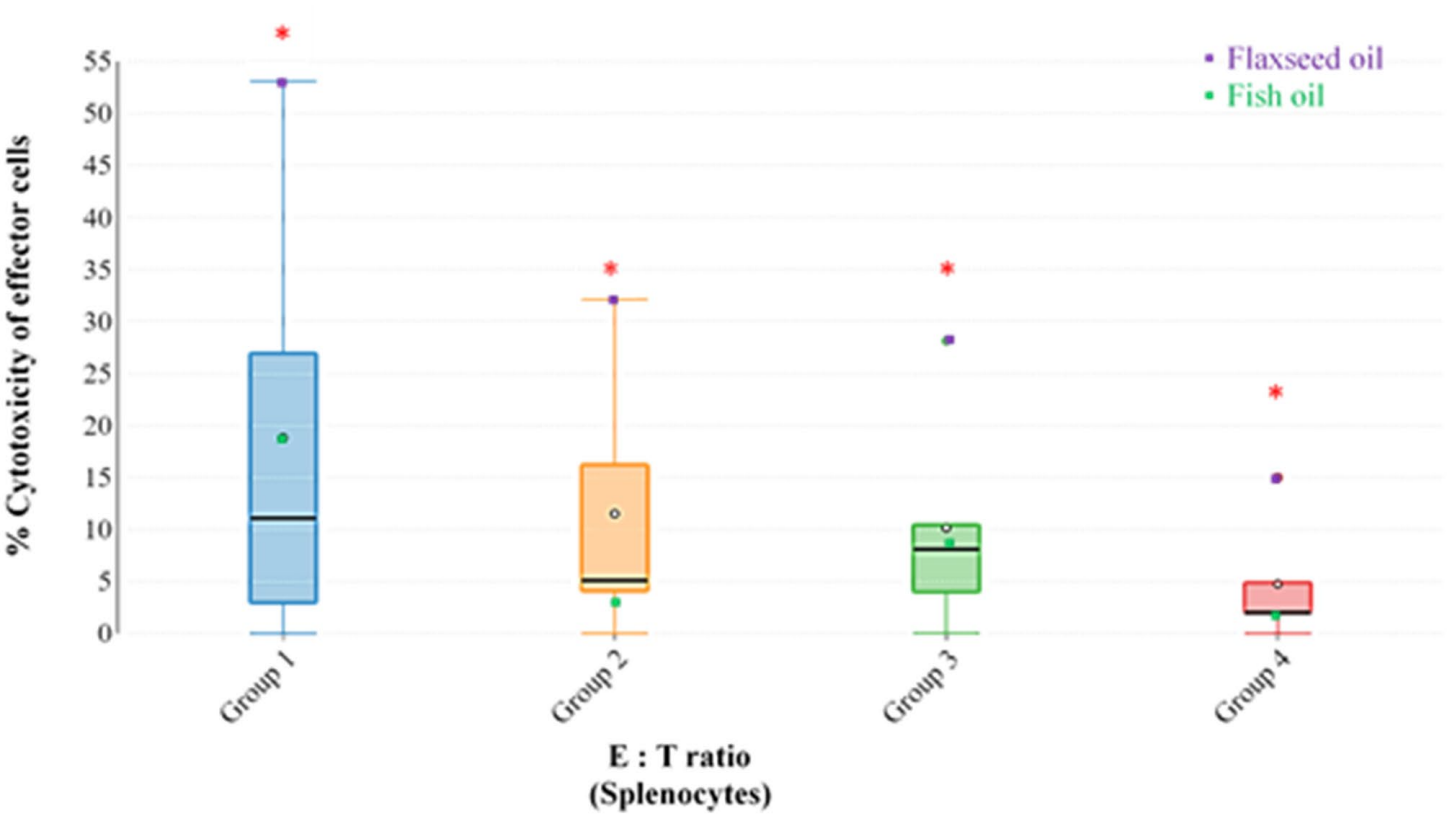
Effect of feeding the experimental dietary treatments on cytotoxic cell activity of splenocytes from 5-week old broilers. Group 1: E:T ratio of 100:1, Group 2: E:T ratio of 50:1, Group 3: E:T ratio of 25:1 and Group 4: E:T ratio of 12.5:1; only statistically significant results obtained from one-way ANOVA are represented by red asterisks (P ≤ 0.05).

**Figure 2:**
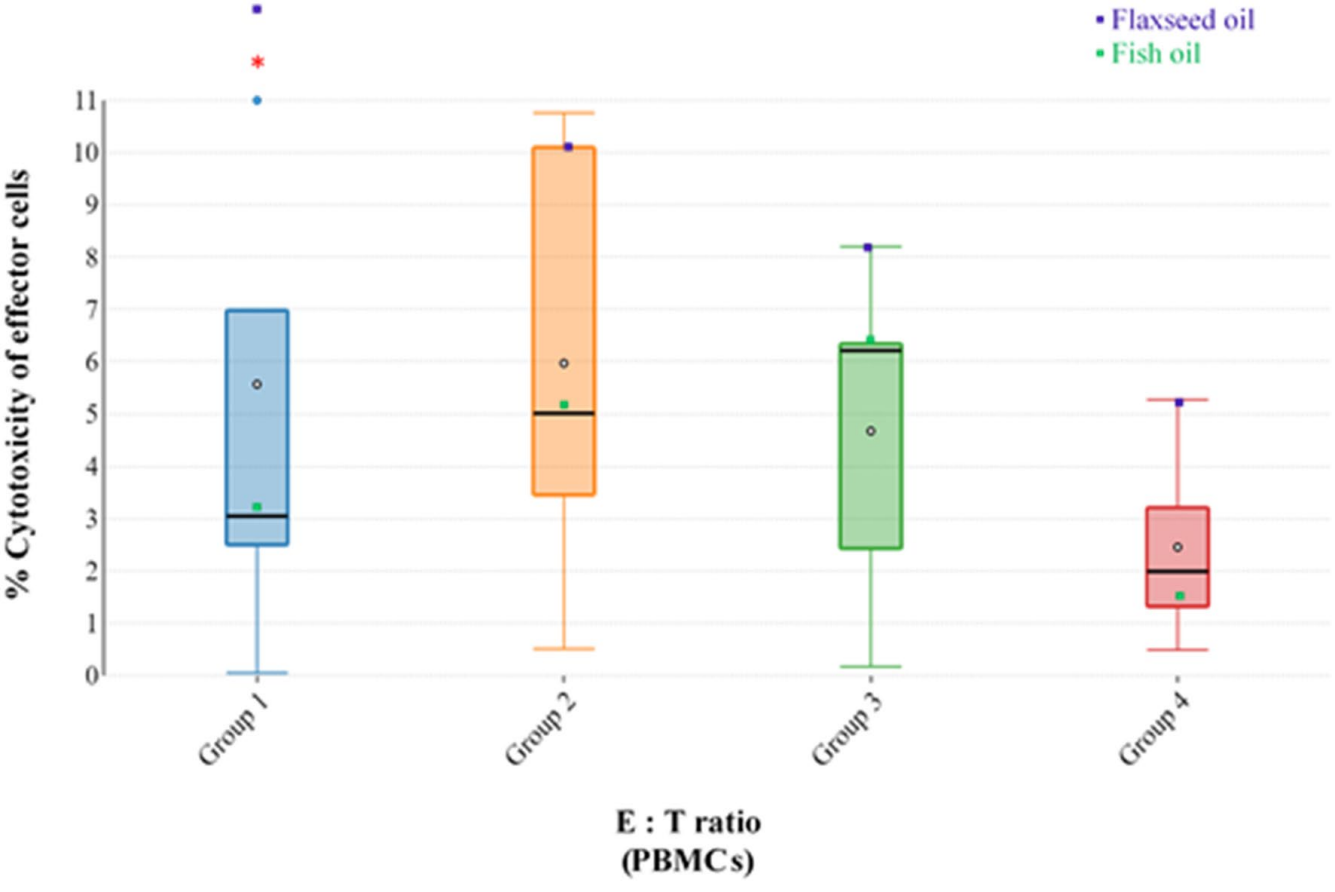
Effect of feeding the experimental dietary treatments on cytotoxic cell activity of PBMCs from 5-week old broilers. Group 1: E:T ratio of 100:1, Group 2: E:T ratio of 50:1, Group 3: E:T ratio of 25:1 and Group 4: E:T ratio of 12.5:1; only statistically significant results obtained from one-way ANOVA are represented by red asterisks (P ≤ 0.05).

The effect of feeding the experimental dietary treatments on cytotoxic cell activity of splenocytes and PBMCs from 5-week old broiler chickens are depicted in Figs. 1 and 2. The results showed that using supplementary flaxseed oil in broiler chickens significantly enhanced cytotoxic cell activity of splenocytes, while FO resulted in the lowest cytotoxic cell activity (Fig. 1). At a ratio of 100:1, cytotoxic cell activity of splenocytes from birds fed the flaxseed oil diet was significantly higher (P < 0.001) than that of broilers fed the control and the FO diets. This effector pattern was the same at lower ratios of effector to target cells with higher cytotoxic cell activity in the flaxseed oil group than the FO and the control group. The same trend was also observed in PBMCs, but the cytotoxic cell activity was generally much lower than that of the splenocytes (Fig. 2). The 100:1 E:T ratio was significantly higher (P < 0.049) for the flaxseed oil diet than control and FO. Tables 6 and 7 show the correlation coefficients for the relationships between % of individual fatty acids in the splenocytes and PBMCS and their cytotoxic cell activity Tables 6 and 7 revealed that the EPA, but not DHA, was consistently negatively correlated with cytotoxic cell activity.

**Table 6:** Correlation coefficients for the relationships between % of individual fatty acids in the splenocytes and their cytotoxic cell activity.

Fatty acid	Cytotoxic cell activity at E:T			
	100		50	
	*r*	*P*	*r*	*P*
C14:00	− 0.100	0.811	0.040	0.999
C16:00	0.010	0.999	0.311	0.440
C16:1n-7	− 0.401	0.201	− 0.192	0.590
C18:00	0.211	0.500	0.191	0.601
C18:1n-9 (oleic)	− 0.191	0.600	0.090	0.821
C18:2n-6 (linoleic)	0.721	0.022	0.645	0.011
C18:3n-6 (γ-linolenic)	− 0.360	0.216	− 0.410	0.212
C18:3n-3 (α-linolenic)	0.400	0.210	0.401	0.200
C20:2	0.655	0.018	0.545	0.040
C20:3n6	0.049	0.900	0.041	0.921
C18:4n-3	− 0.005	0.982	− 0.301	0.355
C20:4n-6 (arachidonic)	0.533	0.040	0.522	0.049
C20:5n-3 (EPA)	− 0.410	0.120	− 0.512	0.082
C22:5n-3	− 0.511	0.070	− 0.412	0.201
C22:6n-3 (DHA)	0.111	0.801	− 0.072	0.856
∑SAT	0.210	0.599	0.300	0.398
∑MUFA	− 0.132	0.610	0.072	0.871
∑PUFA	0.511	0.123	0.445	0.152
∑n-6	0.694	0.019	0.621	0.020
∑n-3	− 0.553	0.060	− 0.461	0.079
∑n-6:∑n-3	0.400	0.192	0.345	0.189

Correlation coefficient (*r*) is Spearman linear rank when data is not normally distributed or Pearson when data is normally distributed.

*EPA* eicosapentaenoic acid, *DHA* docosahexaenoic acid, *DPA* docosapentaenoic acid.

**Table 7:** Correlation coefficients for the relationships between % of individual fatty acids in the PBMCs and their cytotoxic cell activity.

Fatty acid	NK cell activity at E:T			
	100		50	
	*r*	*P*	*r*	*P*
C16:00	− 0.620	0.061	− 0.173	0.699
C16:1n-7	− 0.710	0.022	− 0.232	0.598
C18:00	0.499	0.212	0.130	0.722
C18:1n-9 (oleic)	− 0.698	0.054	− 0.312	0.499
C18:2n-6 (linoleic)	0.798	0.012	0.234	0.559
C18:3n-6 (γ-linolenic)	− 0.493	0.110	0.198	0.722
C18:3n-3 (α-linolenic)	0.678	0.030	0.190	0.571
C20:3n-6	0.199	0.667	0.121	0.731
C18:4 n-3	− 0.251	0.491	0.070	0.851
C20:4n-6 (arachidonic)	0.460	0.144	− 0.150	0.700
C20:5n-3 (EPA)	− 0.761	0.008	− 0.590	0.050
C22:5n-3	− 0.552	0.069	0.200	0.565
C22:6n-3 (DHA)	− 0.170	0.710	− 0.432	0.500
∑SAT	0.009	0.143	0.002	0.999
∑MUFA	− 0.701	0.018	− 0.199	0.699
∑PUFA	0.601	0.087	− 0.281	0.393
∑n-6	0.270	0.399	0.090	0.855
∑n-3	− 0.469	0.148	− 0.400	0.298
∑n-6:∑n-3	0.600	0.091	0.301	0.500

Correlation coefficient (*r*) is Spearman linear rank when data is not normally distributed or Pearson when data is normally distributed.

*EPA* eicosapentaenoic acid, *DHA* docosahexaenoic acid, *DPA* docosapentaenoic acid.

## Discussion

The current study investigated the effect of using flaxseed and FO in the feed ration, production performance parameters, cytotoxic cell activity of splenocytes, and PBMCs of broiler chickens. The production performance parameters analysis showed that the supplemented oils had no impact on the bird's metabolism. These results agree with Kalakuntla et al.^39^, as in their study, dietary supplementation with soy oil, flaxseed oil, and FO also did not influence the chickens' overall performance. In our study, the fatty acid profile of immune cells revealed that enrichment of these cells with n-3 PUFA was successful, with the highest increases in EPA observed in the group of chickens fed FO. On the other hand, this effect was not significant in the flaxseed or the control group. Also, feeding FO resulted in higher docosapentaenoic acid (DPA) (C22:5 n-3) proportion than in the case of flaxseed oil or the control diet (in which EPA would have been formed as a precursor to the DPA). This is because the accumulation of DPA may come from the metabolism of EPA to DPA.

The results revealed that splenocytes exhibited higher cytotoxic cell activity than PBMCs, which is in line with earlier literature^40,41^. The results also showed that feeding broiler chickens on a diet rich in FO resulted in comparatively lower cytotoxic cell activity for both splenocytes and PBMCs than in the flaxseed oil and control groups. Although, in splenocytes, significance (P ≤ 0.05) was observed for all E:T ratios, whereas for PBMCs, significance (P ≤ 0.05) was observed only for 100:1 E:T ratio (Figs. 1, 2). Other studies in animals^30,32,42^ and humans^35,43,44^ are in agreement with the results of the current study.

The inhibitory effects of FO on cytotoxic cell activity, as shown by the correlation analysis in this study, indicate an association between EPA and suppression in cytotoxic cell activity, as was seen with the EPA being negatively correlated with cytotoxic cell activity. On the other hand, there was a positive correlation between n-6 PUFA and cytotoxic cell activity. This could be that the EPA, but not DHA, may be responsible for reducing cytotoxic cell activity in both splenocytes and PBMCs. Supplementing broiler diets with fatty acids can influence the proliferation, maturation, function, and cytokine production of lymphocytes, heterophils, and splenocytes. Earlier studies in literature have observed that supplementing diets with n-3 fatty acids lowered arachidonic acid levels in serum and immune tissues. Still, the levels of EPA and DA increased, which indicated an impact on the immune system. FO is noted to modulate cytokines' production by signal transduction and lymphocytes in immune cells^45^. Polyunsaturated fatty acids are also crucial to eicosanoid formation, which is dependent on the PUFA ratio. It is also critical in the modulation of inflammatory response and duration. N-6 fatty acids (n-3 FAs) possess pro-inflammatory properties, which enhances inflammatory eicosanoid, cytokine production, and immune-suppression, whereas n-3 fatty acids (n-3 FAs) have lesser inflammatory properties by reducing the release of pro-inflammatory eicosanoids and cytokines^46^.

N-3 FAs are characteristic of their immunomodulating properties. Flaxseed is rich plant source of ALA and EPA and DHA are found in fish oil. Poultry do not possess the enzymes required to convert ALA to EPA and DHA and hence are required to be supplemented in their diets. N-3 FAs have the ability to inhibit production of inflammatory mediators like eicosanoids, pro-inflammatory cytokines, chemokines, adhesion molecules etc. Alongside these properties, n-3 FAs also enhance the production of anti-inflammatory cytokines like IL-10 and behave as pro-resolution agents which further act as precursors of pre-resolving mediators like EPA-derived resolvins, DHA-derived resolvins, and protectins and maresins. N-3 FAs are likely to suppress T cell activity through inhibition of Th1 and Th17 differentiation^47^. Abdulwahid and Mudheher^48^ studied the effects of flaxseed oil in combination with a probiotic on broilers' immune response. They concluded that this mixture could enhance the immune response to the Newcastle disease vaccine administered to the chicks. The enrichment of cell membrane with omega-3 FAs is linked to immune cell structure and eicosanoid formation. These FAs have anti-inflammatory properties involving reduction in the release of pro-inflammatory eicosanoids and cytokines. Flaxseed oil supplementation also contributed towards significant blood hematological parameters, which is an indicator of its immunomodulatory properties, which influences many immune mechanisms, modulates phagocytosis, activity of cytotoxic cells, and production of cytokines. Another study used different dietary oil additives on broiler chicks. The oils used were soybean oil, sunflower oil ad fish oil. They used two levels of fish oil, 1 and 2%. Their results showed that fish oil at higher levels (2%) promoted significant effects of the immune system^49^.

Based on the above, the current work suggests that FO's immunosuppressive influence depends mostly on the EPA and the extent to which DHA enrichment of immune tissues. The results are also dependent on the source and concentration of dietary oils and consumption of EPA and DHA levels.

## Methods

### Animal welfare

The committee of the Poultry Production Department at Cairo University approves the procedures executed in the current research. These procedures recommend animal rights and welfare by assuring minimal stress to animals, based on the Ministry of Agriculture's official decrees in Egypt, Decree No. 27 (1967). All methods were carried out in accordance with relevant guidelines and regulations. The study was carried out in compliance with the ARRIVE guidelines.

### Animals and dietary treatments

A total of 255 one-day-old chicks, vaccinated as per the company guidelines, were purchased. Water and feed were provided ad libitum. The temperature was kept at 30 °C for 14 days, and then gradually reduced to 21 °C by 21 days. Upon hatching, all chicks were fed the same basal diet for 21 days. Following this, chicks were randomly assigned to three dietary treatments. The 3-week-old broilers were fed two different sources of n-3 PUFA. The third group continued with the control feed ration.

The broilers were randomly distributed into three batteries or cages (85 birds per battery). Each cage involved five levels, 0.85 m^2^ each. These levels were considered as replicates. Each level accommodated 17 birds, providing a space of 0.05 m^2^ for each bird as recommended by Bell and Weaver^50^. The dietary treatments were randomly distributed between the three batteries. Hence, each treatment was replicated five times. All four cages were located in one room. A total of 20 birds were sacrificed per treatment for sampling, four birds from each replicate.

The sources of n-3 PUFA used were fish oil and flaxseed oil at 50 and 19 g/kg of diet. These levels were determined to match for n-3 PUFA content. The feed rations were prepared according to Cobb 500 guidelines for broilers. The finisher experimental diets' composition is shown in Table 8, and the fatty acid profile of the experimental diets is shown in Table 9.

**Table 8:** Composition of finisher experimental diets^a^ used.

Feed	SO (control)	Flaxseed oil	FO
Wheat	583	583	583
Soybean meal	285	285	285
CaCO_3_	13	13	13
Dicalcium phosphate	10	10	10
Flaxseed oil	0	19	0
Fish oil	0	0	50
Soya oil	32	35	0
Salt	3.5	3.5	3.5
Vitamin/mineral supplement	50	50	50
DL methionine	2	2	2
NaHCO_3_	2	2	2
Lysine	1.5	1.5	1.5
Vitamin E (i.u./kg)	100	100	100
Nutrient composition	Crude protein (5)	Metabolisable energy (kcal/kg)	Fat (g/kg)
	21.03	3105.30	6.002

^a^On as-fed basis.

**Table 9:** Fatty acid composition of the diet mixtures used.

	Fatty acids (wt%)		
	Control	FO	Flaxseed oil
C14:0	0.20	0.91	2.50
C16:0	12.16	11.68	10.75
C16:1	0.40	4.60	1.75
C18:0	4.81	5.40	2.50
C18:1n-9	20.09	20.78	18.55
C18:2n-6 (linoleic)	32.02	43.0	15.00
C18:3n-6 (γ-linolenic)	6.99	0.60	1.50
C18:3n-3 (α-linolenic)	15.55	18.45	16.42
C20:1n-9	0.57	1.0	2.61
C18:4n-3	4.95	0.52	1.20
C20:4n-6 (arachidonic)	5.01	5.28	2.50
C23:0	0.09	1.10	1.20
C20:5n-3 (EPA)	0.21	7.13	0.95
C22:5n-3 (DPA)	0.19	2.20	0.21
C22:6n-3 (DHA)	0.24	8.24	0.19
∑SAT	17.26	19.09	16.95
∑MUFA	21.06	26.38	22.91
∑PUFA	65.16	85.42	37.97
∑n-6	44.02	48.88	19.00
∑n-3	21.14	36.02	18.97
∑n-6:∑n-3	2.08	1.36	1.00

*∑SAT*  sum percentage of saturated fatty acids, *∑MUFA*  sum percentage of monounsaturated fatty acids, *∑PUFA* sum percentage of PUFA, *∑n-6* sum percentage of n-6 PUFA, *∑n-3* sum percentage of n-3 PUFA, *∑n-6:∑n-3 *ratio of ∑n-6 to ∑n-3, *EPA* eicosapentaenoic acid, *DHA* docosahexaenoic acid, *DPA* docosapentaenoic acid, *FO* fish oil.

### Collection of sample

Birds were sacrificed (20 birds per treatment) as per the slaughter principles covered under KS 1174/1999^4^, and blood was collected in heparinized tubes from wing vein using gauge needles from birds from each treatment. Plasma and serum were separated by centrifugation and stored at − 20 °C until analysis. The spleen was removed under aseptic condition and weighed. The spleens were placed in cell culture medium (CCM) on ice. The CCM comprised RMPI-1640 (Sigma-Aldrich, Gillingham, UK) supplemented with 10% fetal calf serum, two mM glutamine, and antibiotics. The spleen was cleaned from all adherent tissues.

### Isolation of peripheral blood mononuclear cells (PBMCs) and splenocytes

The heparinized blood was layered on Lympholyte-H (Cedarlane Laboratories Ltd), and the interface layer with the leukocytes was harvested and suspended in CCM. The cell suspension was then relayered on Lympholyte-H, and the previous step was repeated to remove the unwanted cells. The cells were suspended in CCM. A stainless-steel wire mesh strainer was used to collect the splenocytes from the spleens. The cell suspension was centrifuged and was re-suspended in CCM^51^. Separation of splenocytes from the spleens was performed as described earlier for the blood.

### Fatty acid (FA) analysis

FA profile analyzed using a Hewlett Packard (HP) 6890 series gas chromatography (GC) system (HP, Basingstoke, UK). Approximately 5–9 × 10^7^ cells were used for the analysis as described earlier by Al-Khalifa et al.^8^. The samples were prepared using butylated hydroxytoluene for lipid extraction. For transmethylation, toluene and sulphuric acid in methanol were added to the dried samples. After neutralization, hexane was added, and the solution was transferred to a GC vial. Samples were run against a known fatty acid methyl esters standard solution (47885-U Supelco^®^ 37 component FAME Mix, 10 mg/mL in methylene chloride).

### Cytotoxic cell activity assay

The LSCC-RP9 cell line was used as target cells. 10^5^ of the target cells were maintained in vitro in CCM at 41 °C in an atmosphere containing 5% CO_2_. Cells were serially sub-cultured with CCM every 2 days. Target cells were used within 24–36 h of subculture. Trypan blue dye was used for the determination of cell viability.

The PBMCs and splenocytes (effector cells) were prepared and re-suspended in CCM at 5 × 10^6^ cells/ml. The target cells (5 × 10^6^) of > 95% viability were re-suspended in 1 ml PBS (phosphate buffer saline) and were labelled with diluted 5(6)-carboxyfluorescein diacetate succinimidyl ester (1/50 in PBS CFDA-SE; Fluka brand, from Sigma). Then, this solution (including the cells) was further diluted 1/50 in PBS. The labeled LSCC-RP9 cells were washed, re-suspended in CCM, and calibrated to a final concentration of 5 × 10^4^ cells/ml. A total volume of 200 ml was prepared involving the effector cells and the labeled LSCC-RP9 cells at ratios of 100:1, 50:1, 25:1, and 12.5:1. The solution was incubated for 2 h at 41 °C in an atmosphere with 5% CO_2_. The target cells, whose plasma membranes had been permeabilized by cytotoxic cell activity, were labeled with a red fluorescent DNA dye (propidium iodide, PI). The cell suspension was incubated at 4 °C in the dark for 5 min and then analyzed immediately by FACSCaliburTM flow cytometer (Becton Dickinson). CellQuestTM software was used to acquire and analyze the data. Results of the test were expressed as % cytotoxicity after subtracting % cytotoxicity in the control tubes.

### Statistical analysis

One-way analysis of variance (ANOVA) was used to analyze the overall differences between dietary treatments using Minitab's general linear model. Differences between the means were considered statistically different at P ≤ 0.05. Post-hoc Bonferroni comparison tests were used to determine significantly different means. The data were arcsine transformed to achieve normal distribution. Correlation coefficients for the relationships between % of individual fatty acids in the PBMCs and splenocytes and their cytotoxic cell activity were performed.

## Conclusion

This study showed that flaxseed oil and FO did not impact the production performance of the broilers. Although animal oil source (i.e., FO) compared to plant oil source (flaxseed oil) may have an inhibitory effect on NK activity, this effect may be influenced by other cells of the immune system. No other marker of immune function assessed in the study showed significance by EPA or DHA. Overall enrichment of broiler diets with PUFAs can be beneficial for human health and consumption.
